# On a Fatal Variety of Ulcerative Sore-Throat

**Published:** 1910-10-22

**Authors:** 


### SPECIAL ARTICLE.

### ON A FATAL VARIETY OF ULCERATIVE SORE-THROAT.

That sore-throats are not always easy of diagnosis has become increasingly obvious since bacteriology has become so important a branch of medicine. Not long since we commented upon pneumococcal tonsilitis and laryngitis, mild and severe, ulcerative and non-ulcerative. We have also commented on Vincent's angina which is apt to be mistaken for diphtheria. Quite recently Dr. Goodall has recorded the notes of thirty-five cases of ulcerative sore-throat in which death occurred without the nature of the malady, clearly serious, being obvious. He comes to the conclusion that some at least are examples of scarlatina, in which ulcerative sore-throat has been the main symptom, the rash often escaping observation either from being so transient or from its really being absent altogether. The full paper is published, with the case-notes of the thirty-five patients, in the Annual Report of the Metropolitan Asylums Board for 1909. As he points out, amongst the many cases of disease of the fauces which are erroneously sent to the hospital as diphtheria are some which, though they have been described by previous writers, have not of late attracted much attention in this country, though they form a distinct clinical group and are by no means very rare. The frequency of these cases is variable, but they have been more numerous of late years than formerly. Amongst those sent in as diphtheria are various forms of inflammation and ulceration of the fauces which are apt to be entered in the tables as septic tonsilitis, Vincent's angina, and so forth, and which are different from ordinary forms of acute tonsilitis. For one thing deaths are more frequent amongst them, whereas ordinary tonsilitis very seldom kills.

### Clinical History.

The disease in question begins insidiously like diphtheria, so that in a large proportion of the cases it is already considerably advanced by the time medical aid is sought. The first symptoms may be vomiting, feverishness, loss of appetite, swelling of the neck, usually due to glandular enlargement, and sore-throat, not very severe. The youth of the patient aids the latency of the disease. Only five of the thirty-five cases were five years old and more, and a large number were infants of a few months. The presence of some complication, such as broncho-pneumonia, may divert attention from the faucial affection. When observed from the beginning the latter generally consists of what appears to be diphtheritic membraane on one or both tonsils, occasionally on the uvula and soft palate also, and a watery or muco-purulent discharge from the nose is not infrequently present. It is not surprising that most of the cases are, in the first instance, diagnosed as diphtheria, and they may be accepted as such on admission to the isolation hospital. Thirty out of thirty-four cases, mostly fatal, in which the disease began before the patient was sent to hospital were certified to have diphtheria; the remaining four were certified as scarlet fever, because there was a rash, although the latter was not like that of scarlet fever. There is usually pyrexia and some enlargement of the cervical glands; in the course of two or three days it is noticed that the progress of the disease is not like that of diphtheria; the exudation does not become less, the temperature remains abnormally elevated, and there is no improvement in the general condition of the patient—notwithstanding the fact that diphtheria anti-toxic serum has been given on the supposition that the patient was suffering from diphtheria. It is also to be noticed that the exudation seen on the tonsils and palate does not rest upon a normal or nearly normal mucous membrane, but, on the contrary, covers a surface that is more or less deeply ulcerated. The further course of the disease varies. Not a few of the cases prove fatal; in these the ulcerative process spreads until it may reach the larynx, whilst complications ensue, the commonest being broncho-pneumonia and inflammation and sloughing of the cervical glands, together with the cellular tissues and skin of the neck. Death may be delayed for two or three weeks, or even longer, though in very young patients it may occur within a few days; in cases that are not fatal recovery is tedious, the faucial ulceration being very slow to heal. It is probable, however, that owing to lack of familiarity with the condition the milder cases escape recognition, so that when it is recorded that twenty-five out of Dr. Goodall's thirty-five cases were fatal the mortality is being somewhat overstated.

Rash.

In fifteen cases there was a rash. This most often took the form of a morbiliform erythema, so that measles was suspected. It was seldom difficult to exclude the latter, however, because the distribution, even when universal, was, unlike that of measles, most marked on the extremities. Occasionally the eruption consisted of erythema marginatum, a variety of erythema multiforme, which is never seen in measles. A scarlatiniform erythema is not common.

Complications.

Cervical cellulitis and adenitis, in some cases followed by suppuration or sloughing, occurred in nine cases, thrombosis of the faucial vein and superior longitudinal sinus supervening in one.

Pulmonary complications are also common; they developed in twelve cases. In eleven there was lobular and in one lobar pneumonia. Pleurisy also occurs. In no fewer than nine cases did the ulcerative process extend to the larynx, and doubtless the lung complications were, to a considerable extent, the direct result of this.

Otitis media ensued in six cases, followed in two by suppurative meningitis, and in one by mastoid abscess.

Acute nephritis occurred twice, and so did thrombosis of a cerebral sinus; whilst the following complications were each observed once: stomatitis, glossitis, multiple abscesses and boils, jaundice, enlarged liver and gall-bladder with jaundice, arthritis, and dacryocystitis, whilst in one case there was a distinct relapse.

Illustrative Case.

The following is one of the less severe cases, in which recovery resulted:—

William A., aged nine years, was admitted on October 31, 1908, certified diphtheria. The illness began with a sore-throat on October 27. On admission there was a considerable amount of membrane on both tonsils, which were enlarged, and on the uvula and margin of the soft palate. The cervical glands on each side were enlarged and the breath was very fœtid. On November 2 it was noted that there was definite membrane on the uvula. Cultivations made on that day and the next resulted in a growth of cocci only. For the first few days after admission the temperature varied from 100° F. to 102° F.; it became normal on November 9. On November 4 the exudate present on the uvula and tonsils was described as being pultaceous. There was some painful swelling in front of the left elbow. Another culture made on November 5 again yielded cocci only. On November 6 the nostrils were blocked and there was rhinorrhœa. On the 9th the faucial lesion—tonsils and uvula—was described as being ulcerative. On the 16th there was raw bleeding, ulceration of the margin of the soft palate and adjacent portions of the tonsils. On the 21st there was pultaceous deposit on the uvula. On the 26th the uvula had sloughed away. There was still some ulceration on November 30, but by December 6 there was none. On this day, however, the boy's temperature rose to 101° F., having been normal since November 9; pain in the neck was complained of and the glands became slightly enlarged. On December 8 a foul-smelling rhinorrhœa set in and lasted for some days, and on the 9th the tonsils and soft palate were ulcerated again. A culture was negative so far as diphtheria bacilli were concerned. During the next few days the skin and subcutaneous tissues on the left side of the neck just below the jaw became inflamed, and sloughed. The temperature was raised—99° F. to 102° F.—till December 16. On the 21st the fauces were still ulcerated, but this condition had cleared up by the 28th, when the neck also was nearly well. On the 31st there were redness, swelling, and pain of the right ankle, which continued until January 2. The lad then recovered, and left the hospital on January 29.

Whether this patient gave rise to any cases of scarlet fever in the ward is doubtful. Possibly he did. Two cases did occur, one on January 10 and another on January 20, in patients who had been in the ward some time; but no fewer than three boys, sent in as diphtheria on November 2, December 7, and January 11 respectively were found the next day to be suffering from scarlet fever, and they may have infected the ward.

Bacteriology.

What is the real nature of these cases? Dr. Goodall points out that the question is one of more than scientific interest, for in hospital administration the question arises as to whether they should be isolated, and whether they can be placed safely in diphtheria or scarlet-fever wards. The results of bacteriological examinations are recorded in eighteen of the cases: In fourteen diphtheria bacilli could not be found; in one they were present, whilst in three bacilli resembling, but not certainly, diphtheria bacilli were detected. In only two cases were smear preparations examined, and in neither of them were the fusiform bacilli and the spirilla described by Vincent to be found; besides, Vincent's angina is seldom fatal. The bacteriological findings confirm the clinical evidence that these cases are not diphtheria; even in the few instances in which Klebs Loeffler bacilli are found they may in part be due to subsequent infection.

Are these Cases Scarlet Fever?

Clinically these cases resemble scarlet fever much more closely than they do diphtheria. It is important, therefore, to inquire into their relationship to scarlatina.

In the first place, Dr. Goodall points out that not a single one of his cases presented at all markedly the punctate erythema usually seen in scarlet fever; and this though they were all, without exception, cases that would be styled severe. In only three of them was there a punctate rash at all, and in them it was indefinite and transient. On the other hand, there was acute nephritis in two cases, a high proportion, which certainly suggests scarlet fever. A certain amount of desquamation was noticed in seven, in all but one of which there had been an erythema; in only one case, however, was peeling at all general. In this connection, it should be borne in mind that not a few of the patients died before peeling was likely to begin. Perforation of one of the anterior pillars of the soft palate, which Dr. Goodall showed years ago to be suggestive of scarlet fever, occurred in three cases, and the faucial lesions generally were such as are found in cases of severe scarlatina anginosa.

Other evidence of scarlet fever might be afforded by the occurrence of cases that may have been infected from those under discussion. Using the same numbers as Dr. Goodall does in referring to his cases, the facts respecting this point are as follows: Cases 1 to 10 and case 32 were treated in isolation rooms, and therefore had no opportunity of infecting other patients. Cases 12 to 18 and case 30 were treated in diphtheria wards for various periods as follows: 23, 29, 15, 4, 15, 6, 7, and 1 day respectively, till they died. Though they were all severe cases, requiring constant attention from the nursing staff, no other patients caught scarlet fever. Cases 19 to 27 and case 31 were possibly connected with the outbreak of scarlet-fever cases, though the evidence was on the whole against this in cases 19 to 23 and case 31. In cases 24 to 27 there were somewhat stronger reasons for supposing that the patient had given rise to scarlet fever in another patient, though even in these the evidence is far from conclusive. Case 27 was placed in a diphtheria ward on admission on September 30; on October 9 another patient developed scarlet fever, upon which case 27 was transferred to a scarlet-fever ward, where she remained till she left the hospital on December 11. Cases 28 and 29 were also treated in a scarlet-fever ward for a considerable period without contracting the disease. In cases 30, 31, 32, 33, and 34 one or more other members of the family were attacked by scarlet fever at about the same time that the patient fell ill. Case 31 was kept in a diphtheria ward for thirty-four days, and possibly introduced scarlet fever into that ward. Case 32 was isolated. Cases 33 and 34 were treated in scarlet-fever wards and did not contract that disease. A brother and sister of case 30, a sister of case 31, a brother of case 32, a sister of case 33, and a sister of case 34 had undoubted scarlet fever —a fact which may be taken as evidence that cases 30 to 34 were also instances of that malady. Case 35 underwent undoubted attacks of both diphtheria and scarlet fever before he was, when convalescent from the latter, attacked with the ulcerative disease of the fauces which caused his death.

Dr. Goodall concludes from the evidence that some at least of these cases are really scarlet fever, though others are perhaps not. If all of them are to be regarded as scarlatinal, then absence of the punctate rash in scarlet-fever cases must be allowed to be commoner than has hitherto been supposed. It is worth while noting that the disease, whatever it may be, may attack more than one member of a family simultaneously.

### Historical.

Dr. Goodall points out that both Fothergill and Huxham described "ulcerous sore throats" and "angina maligna" as long ago as the middle of the eighteenth century. Huxham's "Essay on Fevers and a Dissertation on the Malignant, Ulcerous Sore Throat" had reached its sixth edition by 1769, and in it he expresses the view that some of the cases of scarlet fever mentioned by Morton were truly not unlike some of the cases of ulcerous sore throat recorded by himself. At the same time, not all the cases observed by those writers were alike; nevertheless, there appears to be little, if any, difference between the severe kinds of "angina" with which those observers were familiar and those described by Dr. Goodall to-day. The cases are perhaps less frequent now in a severe form, but in their clinical aspects they are just the same, and, largely owing to there being no bacteriological basis for their diagnosis, it is no less difficult now than it was then to be sure whether they are or are not really scarlet fever.

